# Factors Associated With In‐Hospital Mortality and Length of Stay Among Hospitalised Patients Receiving Carbapenem Therapy: A Retrospective Cohort Study

**DOI:** 10.1155/ipid/7227340

**Published:** 2026-10-19

**Authors:** Abdullah Faiz Zaihan, Dhivyaa K. Ganesan, Chia Siang Kow

**Affiliations:** ^1^ Department of Pharmacy Shah Alam Hospital Shah Alam Selangor Malaysia; ^2^ School of Pharmacy IMU University Kuala Lumpur Malaysia imu.edu.my; ^3^ School of Applied Sciences University of Huddersfield Huddersfield UK hud.ac.uk

**Keywords:** antimicrobial stewardship, carbapenems, Firth regression, in-hospital mortality, length of stay, median regression, observational study

## Abstract

**Background:**

Patients receiving carbapenems are clinically heterogeneous and have severe infections and multiple comorbidities. Evidence on factors associated with mortality and length of stay (LOS) in this treatment‐defined population remains limited.

**Methods:**

This retrospective cohort study included adult hospital episodes involving at least one carbapenem dose at Hospital Shah Alam during 2025. Firth penalised logistic regression was used for in‐hospital mortality, and median regression with 2000 bootstrap resamples was used for right‐skewed LOS. Covariates were selected a priori. Carbapenem continuation beyond 72 h was treated as a postbaseline characteristic and was not included in outcome models.

**Results:**

Among 112 eligible episodes involving 107 patients, 38 deaths occurred (33.9%); median LOS was 19.0 days (IQR: 10.8–28.0). Older age (adjusted OR: 1.41 per 10 years, 95% CI: 1.07–1.92) and definitive indication at carbapenem initiation (adjusted OR: 0.28, 95% CI: 0.10–0.70) were associated with in‐hospital mortality. Hospital‐acquired infection was associated with a longer adjusted median LOS (median difference 8.63 days, 95% bootstrap CI: 0.50–14.44). Definitive indication was not clearly associated with LOS after adjustment (median difference −5.54 days, 95% bootstrap CI: −12.00–4.62).

**Conclusions:**

Older age and definitive indication at carbapenem initiation were associated with in‐hospital mortality, while hospital‐acquired infection was associated with longer median LOS. The treatment‐indication association should not be interpreted causally because residual confounding, particularly by acute illness severity and indication, remains likely.

## 1. Introduction

Carbapenem antibiotics are among the most potent broad‐spectrum antimicrobial agents and are widely used in hospital settings for the management of severe infections, particularly those caused by multidrug‐resistant Gram‐negative organisms. Their use is typically reserved for patients with complex clinical profiles, including those with multiple comorbidities, healthcare‐associated infections and severe or rapidly progressing disease [1, 2]. As a result, patients receiving carbapenem therapy often represent a high‐risk population with a substantial burden of illness and an increased likelihood of adverse clinical outcomes.

In‐hospital mortality and prolonged length of stay (LOS) are key outcomes in this population, reflecting both disease severity and the effectiveness of clinical management. Previous studies have consistently demonstrated that older age and comorbidity burden are associated with increased mortality in patients with severe infections [3–5]. Similarly, healthcare‐associated infections, delayed clinical response, and complications during hospitalisation are recognized contributors to prolonged hospital stay [6–9]. However, these outcomes are multifactorial and may be influenced by a combination of patient characteristics, infection‐related factors and treatment decisions.

In addition to patient and infection‐related factors, treatment‐related variables may play an important role in determining outcomes [10–12]. In clinical practice, carbapenem therapy may be initiated empirically in patients with suspected severe infections or used as definitive therapy based on microbiological confirmation [13, 14]. These treatment strategies reflect different stages of clinical decision‐making and may be associated with varying disease severity, pathogen profiles and responses to therapy. However, the independent contribution of such treatment‐related factors to clinical outcomes remains unclear, particularly in real‐world settings.

Antimicrobial stewardship programmes have emphasised the importance of optimising antibiotic use, not only to reduce antimicrobial resistance but also to improve patient outcomes [15]. Despite this, most stewardship efforts have focused on prescribing appropriateness and antibiotic utilization, with comparatively less emphasis on understanding how clinical and treatment‐related variables interact to influence outcomes such as mortality and LOS among patients receiving broad‐spectrum antibiotics.

Furthermore, evidence from real‐world clinical settings remains limited, especially in Malaysian healthcare systems where patterns of infection, antimicrobial resistance and prescribing practices may differ from those reported in international studies. Evaluating outcomes within a local cohort is, therefore, important to provide context‐specific insights that can inform clinical decision‐making and stewardship strategies.

Therefore, this study aimed to evaluate baseline demographic, clinical, infection‐related and treatment‐indication factors associated with in‐hospital mortality and LOS among hospitalised adults receiving carbapenem therapy at a secondary hospital in Malaysia.

## 2. Methods

### 2.1. Study Design and Setting

A retrospective cohort study was conducted at Hospital Shah Alam, a secondary care public hospital in Selangor, Malaysia. The study considered all hospital episodes involving carbapenem therapy between 1 January 2025 and 31 December 2025.

### 2.2. Study Population

All consecutive hospital episodes involving patients aged 18 years or older who received at least one dose of meropenem, ertapenem or another documented carbapenem exposure were eligible. Episodes were identified through pharmacy dispensing records and verified against electronic and paper medical records.

Five patients contributed two episodes each. Repeated admissions were analysed as separate episodes because exposures and outcomes were episode specific; a sensitivity analysis retained only each patient’s first eligible episode.

### 2.3. Data Collection and Variables

Data were extracted retrospectively using a standardised data‐collection form. Baseline variables were defined using information documented at or before carbapenem initiation.

• Demographic and comorbidity variables comprised age, sex, ethnicity, smoking status, hypertension, diabetes mellitus, chronic kidney disease, coronary artery disease/ischaemic heart disease, heart failure, stroke, atrial fibrillation, dyslipidaemia and chronic respiratory disease. A count of the nine recorded comorbidities was used to represent chronic comorbidity burden in adjusted models.
• Clinical variables comprised baseline estimated glomerular filtration rate (eGFR) and lactate where available. eGFR values recorded as > 90 mL/min/1.73 m^2^ were conservatively coded as 90. Lactate was available for only 9 of 112 episodes and was therefore summarised descriptively but not entered into regression models.
• The mutually exclusive primary infection categories were respiratory infection, urinary tract infection, bloodstream infection/central‐line‐associated bloodstream infection, intra‐abdominal/general surgery infection, sepsis without a documented anatomical source and other infection types. Category assignment followed the primary infection source or diagnosis documented by the treating team. Sepsis without a documented anatomical source was used when sepsis was documented but no anatomical focus was identified in the available record; bloodstream infection/central‐line‐associated bloodstream infection was used when the bloodstream or central venous catheter was documented as the primary infection source. For regression, respiratory infection was represented separately from all other categories because pulmonary infection is a clinically distinct syndrome in carbapenem‐treated inpatients, particularly in hospital‐acquired pneumonia, and respiratory infection was the most frequent infection category in this cohort. Modelling each infection category separately would also have introduced several sparsely populated parameters given the modest number of deaths. The pooled nonrespiratory comparator was, therefore, used for model parsimony and does not imply clinical equivalence among nonrespiratory infection types. Acquisition status was abstracted as community‐ or hospital‐acquired. Hospital‐acquired infection was defined as infection developing at least 48 h after admission and not present or incubating at admission.
• Treatment variables comprised the carbapenem type, completion of the antimicrobial restriction/request form and indication category at carbapenem initiation. Empirical therapy was defined as carbapenem initiation before identification and susceptibility confirmation of a causative organism. Definitive therapy was defined as carbapenem initiation or switching to a carbapenem when organism identification and susceptibility findings were already available and informed selection. Patients who began carbapenem empirically and only later received confirmatory microbiology remained classified as empirical because classification was fixed at carbapenem initiation. Although definitive therapy could include switching from another antibiotic to a carbapenem after susceptibility results became available, time‐resolved antibiotic escalation and de‐escalation were not predefined study variables and were, therefore, not analysed.
• Carbapenem continuation beyond 72 h was reported only as a postbaseline stewardship characteristic. It was not included in mortality and length‐of‐stay models because classification required survival and continued observation after treatment initiation, creating potential immortal time bias for mortality and reverse causality for LOS [16].

### 2.4. Outcome Measures

The study outcomes were as follows:

1. In‐hospital mortality, defined as death from any cause during the index hospital episode.
2. LOS, defined as the number of days from hospital admission to discharge or death.

### 2.5. Statistical Analysis

Baseline characteristics were summarised using medians with interquartile ranges (IQRs) for continuous variables and frequencies with percentages for categorical variables. The distribution of LOS was assessed using its range, skewness and the Shapiro–Wilk test. Residual normality and homoscedasticity in the originally specified linear model were assessed using the Shapiro–Wilk and Breusch–Pagan tests, respectively.

Adjusted models used covariates selected a priori for clinical relevance and potential confounding rather than univariable *p* values: age, sex, baseline eGFR, number of recorded comorbidities, acquisition source, respiratory infection and definitive versus empirical indication at carbapenem initiation.

In‐hospital mortality was analysed using Firth penalised logistic regression to reduce small‐sample maximum‐likelihood bias with 38 outcome events [17]. Associations are reported as adjusted odds ratios (ORs) with profile‐likelihood 95% confidence intervals (CIs). Multicollinearity was assessed using variance inflation factors (VIFs). Discrimination and fit were summarised using the c‐statistic with a 2000‐resample bootstrap CI, Brier score and Hosmer–Lemeshow test.

Because LOS and linear‐model residuals were markedly right skewed, LOS was analysed using median (quantile) regression [18]. Adjusted coefficients represent differences in median LOS in days. Percentile 95% CIs and two‐sided *p* values were estimated using 2000 pairwise bootstrap resamples; bootstrap convergence and median‐regression pseudo‐*R* ^2^ were recorded.

The primary models used complete cases. All prespecified model variables and both outcomes were complete in the 112 eligible episodes; no imputation was performed. Smoking status recorded as ‘unknown’ was retained as a descriptive category rather than treated as missing. A sensitivity analysis of mortality retained the first eligible episode for each patient. Statistical significance was defined as a two‐sided *p* value < 0.05.

## 3. Results

### 3.1. Cohort and Baseline Characteristics

The analysis included 112 adult episodes among 107 unique patients; five patients contributed two episodes each. Median age was 58.0 years (IQR 48.0–68.0), and 61 episodes (54.5%) involved male patients (Table 1).

**TABLE 1:** Characteristics of adult hospital episodes involving carbapenem therapy.

Characteristic	Overall (*N* = 112)
Demographics	
Age (years)	58.0 (48.0–68.0)
Male sex	61 (54.5)
Ethnicity	
Malay	63 (56.3)
Chinese	12 (10.7)
Indian	22 (19.6)
Other	15 (13.4)
Smoking status	
Current smoker	14 (12.5)
Former smoker	5 (4.5)
Never smoker	12 (10.7)
Unknown	81 (72.3)
Comorbidities	
Diabetes mellitus	71 (63.4)
Hypertension	75 (67.0)
Chronic kidney disease	28 (25.0)
Coronary artery disease/ischaemic heart disease	31 (27.7)
Heart failure	11 (9.8)
Stroke/cerebrovascular accident	15 (13.4)
COPD/asthma	6 (5.4)
Atrial fibrillation	7 (6.3)
Dyslipidaemia	46 (41.1)
Recorded comorbidity count	3.0 (1.0–4.0)
Baseline clinical parameters	
Baseline eGFR (mL/min/1.73 m^2^)	52.0 (24.5–81.0)
Baseline lactate (mmol/L) (*n* = 9)	1.32 (0.88–1.98)
Source of infection acquisition	
Community‐acquired infection	19 (17.0)
Hospital‐acquired infection	93 (83.0)
Primary infection category	
Respiratory infection	50 (44.6)
Urinary tract infection	24 (21.4)
Bloodstream infection/CRBSI	19 (17.0)
Sepsis without documented anatomical source	8 (7.1)
Intra‐abdominal/general surgery infection	6 (5.4)
Other infection type	5 (4.5)
Treatment/stewardship characteristics	
Antimicrobial restriction/request form completed at initiation	37 (33.0)
Category of indication at carbapenem initiation	
Empirical indication	53 (47.3)
Definitive indication	59 (52.7)
Type of carbapenem	
Meropenem	98 (87.5)
Ertapenem	10 (8.9)
Mixed carbapenem exposure	4 (3.6)
Carbapenem continued beyond 72 h	75 (67.0)

*Note:* Continuous variables, including baseline eGFR and lactate, are presented as median (interquartile range); categorical variables are presented as *n* (%). eGFR values recorded as > 90 mL/min/1.73 m^2^ were coded as 90. Lactate was missing for 103 episodes. Infection categories were mutually exclusive. Continuation beyond 72 h is descriptive and was not included in outcome models. CRBSI, central‐line‐associated bloodstream infection.

Abbreviations: COPD, chronic obstructive pulmonary disease; eGFR, estimated glomerular filtration rate.

Ethnicity was Malay in 63 episodes (56.3%), Chinese in 12 (10.7%), Indian in 22 (19.6%) and other in 15 (13.4%). Hypertension (67.0%) and diabetes mellitus (63.4%) were the most frequent recorded comorbidities. Smoking status was recorded as unknown in 81 episodes (72.3%).

Median baseline eGFR was 52.0 mL/min/1.73 m^2^ (IQR: 24.5–81.0). Lactate was available for nine episodes (8.0%), with a median of 1.32 mmol/L (IQR 0.88–1.98). Hospital‐acquired infections accounted for 93 episodes (83.0%), and respiratory infections were the largest mutually exclusive primary infection category (*n* = 50, 44.6%).

At carbapenem initiation, 59 episodes (52.7%) were classified as definitive and 53 (47.3%) as empirical. Meropenem was used in 98 episodes (87.5%), ertapenem in 10 (8.9%) and mixed carbapenem exposure in 4 (3.6%). Carbapenem was continued beyond 72 h in 75 episodes (67.0%).

### 3.2. Factors Associated With In‐Hospital Mortality

In‐hospital death occurred in 38 of 112 episodes (33.9%). The Firth penalised logistic model included all 112 episodes and the seven prespecified covariates (Table 2).

**TABLE 2:** Firth penalised logistic regression for in‐hospital mortality.

Variable	Comparison/scale	Adjusted OR	95% CI	*p* value
Age	Per 10‐year increase	1.41	1.07–1.92	0.015
Sex	Male vs. female	2.16	0.89–5.49	0.088
Baseline eGFR	Per 10 mL/min/1.73 m^2^	0.99	0.85–1.16	0.925
Recorded comorbidities	Per additional condition	0.84	0.65–1.09	0.187
Infection acquisition	Hospital vs. community	0.55	0.17–1.75	0.303
Respiratory infection	Yes vs. all other categories	1.43	0.60–3.46	0.421
Indication at initiation	Definitive vs. empirical	0.28	0.10–0.70	0.006

*Note: N* = 112 hospital episodes; 38 deaths. Covariates were selected a priori. Adjusted ORs and profile‐likelihood 95% CIs are shown. Carbapenem continuation beyond 72 h was excluded because it was a postbaseline characteristic.

Abbreviations: CI, confidence interval; eGFR, estimated glomerular filtration rate; OR, odds ratio.

Older age was associated with higher odds of in‐hospital mortality (adjusted OR: 1.41 per 10‐year increase, 95% CI: 1.07–1.92; *p* = 0.015). Definitive indication at carbapenem initiation was associated with lower odds of mortality than empirical indication (adjusted OR: 0.28, 95% CI: 0.10–0.70; *p* = 0.006). This treatment‐indication association is observational and does not estimate a causal treatment effect.

No clear adjusted associations were observed for sex, baseline eGFR, recorded comorbidity count, hospital‐acquired infection or respiratory infection. The maximum VIF was 1.40. The model c‐statistic was 0.760 (95% bootstrap CI: 0.661–0.854), the Brier score was 0.183, and the Hosmer–Lemeshow test did not indicate gross lack of fit (*p* = 0.145).

In the sensitivity analysis restricted to each patient’s first eligible episode (*n* = 107; 37 deaths), the association for definitive indication was similar (adjusted OR: 0.29, 95% CI: 0.11–0.74; *p* = 0.009).

### 3.3. Factors Associated With LOS

Median LOS was 19.0 days (IQR: 10.8–28.0; range 3–102), with marked right skewness (2.12). Both LOS values and residuals from the originally specified linear model were nonnormally distributed (Shapiro–Wilk *p* < 0.001). The Breusch–Pagan test did not detect heteroscedasticity (*p* = 0.390); nevertheless, marked nonnormality supported use of median regression.

Hospital‐acquired infection was associated with an 8.63‐day longer adjusted median LOS than community‐acquired infection (95% bootstrap CI: 0.50–14.44; *p* = 0.035) (Table 3). No clear adjusted LOS associations were observed for age, sex, baseline eGFR, recorded comorbidity count, respiratory infection or definitive indication at carbapenem initiation.

**TABLE 3:** Median regression for length of stay.

Variable	Comparison/scale	Adjusted median difference (days)	95% CI	*p* value
Age	Per 10‐year increase	0.57	−1.88 to 2.95	0.627
Sex	Male vs. female	5.34	−2.40 to 10.21	0.233
Baseline eGFR	Per 10 mL/min/1.73 m^2^	−0.19	−1.41 to 1.58	0.879
Recorded comorbidities	Per additional condition	0.67	−1.55 to 2.28	0.656
Infection acquisition	Hospital vs. community	8.63	0.50 to 14.44	0.035
Respiratory infection	Yes vs all other categories	−2.10	−8.91 to 6.12	0.686
Indication at initiation	Definitive vs. empirical	−5.54	−12.00 to 4.62	0.521

*Note: N* = 112 hospital episodes. Coefficients represent adjusted differences in median length of stay in days. Percentile 95% CIs and *p* values were obtained from 2000 pairwise bootstrap resamples. Carbapenem continuation beyond 72 h was excluded because of reverse causality.

Abbreviations: CI, confidence interval; eGFR, estimated glomerular filtration rate.

All 2000 bootstrap median‐regression fits converged. The model pseudo‐*R* ^2^ was 0.056, indicating limited improvement in model fit relative to an intercept‐only median‐regression model. This pseudo‐*R* ^2^ is not directly comparable with the *R* ^2^ from ordinary least‐squares regression and should not be interpreted as the percentage of total LOS variance explained.

## 4. Discussion

In this retrospective cohort of 112 adult hospital episodes involving carbapenem therapy, older age and definitive indication at carbapenem initiation were associated with in‐hospital mortality, while hospital‐acquired infection was associated with longer median LOS.

The association between older age and mortality is consistent with evidence from patients with severe infection and sepsis, in whom reduced physiological reserve, frailty, comorbidity and vulnerability to organ dysfunction contribute to poorer outcomes [3, 4, 19–21]. The effect estimate in this study should nevertheless be interpreted within the broad uncertainty produced by the modest number of deaths.

Definitive indication at carbapenem initiation was associated with lower mortality, but this finding does not establish that definitive carbapenem therapy reduced mortality. Classification as definitive required microbiological information to be available when carbapenem was selected and may, therefore, identify a different clinical pathway and patient group. Disease severity, source control, pathogen and susceptibility profiles, appropriateness and timing of preceding antibiotics and early clinical response could influence both treatment classification and outcome [10, 11, 22]. In addition, because empirical classification was fixed at carbapenem initiation, the empirical group could include patients who deteriorated or died before organism identification and susceptibility results became available. Such patients would remain classified as empirical and could enrich that group with rapidly fatal illness, potentially contributing to the observed mortality association. The timing of microbiological results relative to clinical outcome was not a predefined study variable, so this potential mechanism could not be quantified. Residual confounding by indication, therefore, remains a substantial alternative explanation.

Continuation beyond 72 h was deliberately excluded from both adjusted models. Patients classified as continuing therapy must remain alive and under observation long enough to acquire that status, whereas early death or transfer may prevent classification as continued therapy. Treating this postbaseline status as a conventional predictor can, therefore, introduce immortal time bias for mortality [16]. For LOS, prolonged hospitalisation itself creates more opportunity for therapy to continue, producing reverse causality. Mortality within 72 h of carbapenem initiation was not collected as a predefined study variable and could not be reliably ascertained for all patients; it was, therefore, not analysed separately. Accordingly, a 72‐h landmark or time‐dependent outcome analysis was not performed.

Hospital‐acquired infection was associated with a longer adjusted median LOS, consistent with previous reports linking healthcare‐associated infection with additional treatment, monitoring, source‐control requirements and hospital resource use [6, 7, 23, 24]. However, only 19 episodes were classified as community‐acquired, resulting in an imbalanced comparison and a wide CI. The estimate should, therefore, be viewed as an association that may also reflect unmeasured illness complexity. Although the median‐regression pseudo‐*R* ^2^ was low (0.056), this statistic reflects limited overall model fit rather than a literal estimate that 5.6% of LOS variance was explained. The adjusted 8.63‐day difference in median LOS may nevertheless be clinically and operationally meaningful, particularly for hospital bed use and resource planning, while substantial unexplained heterogeneity in LOS remains.

Definitive indication was not clearly associated with median LOS in the revised model. Accordingly, the present data should not be used to infer that microbiologically directed carbapenem therapy shortens hospitalisation. Evidence supporting antimicrobial reassessment and de‐escalation comes from studies designed around a clearly timed stewardship decision, including target‐trial and propensity‐based analyses [25–27], rather than from the treatment‐indication comparison used here.

Clinically, the findings support careful risk assessment in older adults and reinforce the substantial resource burden associated with hospital‐acquired infection. They also emphasise the value of obtaining microbiological data promptly. However, this study does not determine whether carbapenem continuation, discontinuation, escalation or de‐escalation changes mortality or LOS, and it should not be used as a treatment‐selection rule.

Several limitations should be considered. The retrospective design permits incomplete documentation and misclassification. Acute illness severity variables—including ICU admission, septic shock, vasopressor use, mechanical ventilation, organ dysfunction and validated severity scores—were not systematically captured. Lactate was available in only 9 of 112 episodes. Mortality within 72 h, timing of microbiological results relative to clinical outcomes and time‐resolved antibiotic escalation or de‐escalation were not predefined study variables. Consequently, the adjusted models could not adequately control for acute severity or fully reconstruct antibiotic treatment pathways, and the definitive‐indication association is particularly susceptible to residual confounding by indication.

The modest sample and 38 deaths limited model complexity and precision; Firth regression reduced small‐sample estimation bias but could not eliminate overfitting or unmeasured confounding. LOS ended at either discharge or death, so early death may shorten observed LOS. Five patients contributed two episodes although a first‐episode sensitivity analysis produced a similar mortality estimate for definitive indication. eGFR values recorded as > 90 were top‐coded at 90. Finally, this single‐centre secondary‐hospital cohort may not be generalisable to other patient populations or carbapenem‐use pathways.

Despite these limitations, the revised analysis distinguishes baseline factors from postbaseline stewardship status, uses methods appropriate to the event count and skewed LOS distribution and provides transparent estimates that can inform the design of larger studies with time‐resolved treatment and severity data.

## 5. Conclusion

Among hospitalised adults receiving carbapenem therapy, older age and definitive indication at carbapenem initiation were associated with in‐hospital mortality, while hospital‐acquired infection was associated with longer median LOS.

These findings describe associations rather than causal treatment effects. Larger studies incorporating acute illness severity, microbiological timing, source control and time‐varying antibiotic exposure are required before treatment‐related conclusions can be drawn.

## Author Contributions

Abdullah Faiz Zaihan and Chia Siang Kow contributed to the conception and design of the study. Abdullah Faiz Zaihan and Dhivyaa K. Ganesan contributed to data acquisition, data management and verification. Abdullah Faiz Zaihan performed the data analysis and drafted the initial manuscript. Dhivyaa K. Ganesan contributed to data interpretation and manuscript revision. Chia Siang Kow provided study supervision, interpretation of findings and critical revision of the manuscript for important intellectual content.

## Funding

This research received no specific grant from any funding agency in the public, commercial or not‐for‐profit sectors.

## Disclosure

All authors reviewed and approved the final version of the manuscript and agreed to be accountable for the integrity and accuracy of the work.

All authors meet the ICMJE authorship criteria.

## Ethics Statement

This retrospective study was registered with the National Medical Research Register (NMRR ID‐26‐00503‐F6F) and approved by the Medical Research and Ethics Committee (MREC), Ministry of Health Malaysia.

## Consent

The authors have nothing to report.

## Conflicts of Interest

The authors declare no conflicts of interest.

## Data Availability

The data that support the findings of this study are available from the corresponding author upon reasonable request.

## References

[1] Tamma P. D. , Aitken S. L. , Bonomo R. A. , Mathers A. J. , van Duin D. , and Clancy C. J. , Infectious Diseases Society of America 2022 Guidance on the Treatment of Extended-Spectrum β-Lactamase Producing Enterobacterales (ESBL-E), Carbapenem-Resistant Enterobacterales (CRE), and *Pseudomonas aeruginosa* With Difficult-to-Treat Resistance (DTR-P. aeruginosa), Clinical Infectious Diseases. (2022) 75, no. 2, 187–212, 10.1093/cid/ciac268. PMC9890506 35439291

[2] Papp-Wallace K. M. , Endimiani A. , Taracila M. A. , and Bonomo R. A. , Carbapenems: Past, Present, and Future, Antimicrobial Agents and Chemotherapy. (2011) 55, no. 11, 4943–4960, 10.1128/AAC.00296-11. PMC3195018 21859938

[3] Yang Y. , Yang K. S. , Hsann Y. M. , Lim V. , and Ong B. C. , The Effect of Comorbidity and Age on Hospital Mortality and Length of Stay in Patients With Sepsis, Journal of Critical Care. (2010) 25, no. 3, 398–405, 10.1016/j.jcrc.2009.09.001. 19836195

[4] Martin-Loeches I. , Guia M. C. , Vallecoccia M. S. et al., Risk Factors for Mortality in Elderly and Very Elderly Critically Ill Patients With Sepsis: A Prospective, Observational, Multicenter Cohort Study, Annals of Intensive Care. (2019) 9, no. 1, 10.1186/s13613-019-0495-x. PMC6362175 30715638

[5] Shankar-Hari M. , Harrison D. A. , Ferrando-Vivas P. , Rubenfeld G. D. , and Rowan K. , Risk Factors at Index Hospitalization Associated With Longer-Term Mortality in Adult Sepsis Survivors, JAMA Network Open. (2019) 2, no. 5, 10.1001/jamanetworkopen.2019.4900. PMC6547123 31150081

[6] Stewart S. , Robertson C. , Pan J. et al., Impact of Healthcare-Associated Infection on Length of Stay, Journal of Hospital Infection. (2021) 114, 23–31, 10.1016/j.jhin.2021.02.026. 34301393

[7] Cai Y. , Lo J. J. , Venkatachalam I. et al., The Impact of Healthcare Associated Infections on Mortality and Length of Stay in Singapore-A Time-Varying Analysis, Infection Control and Hospital Epidemiology. (2020) 41, no. 11, 1315–1320, 10.1017/ice.2020.304. 32665057

[8] Zeng H. , Yang C. , Jiang M. et al., Predicting Length of Hospital Stay in Community-Acquired Pneumonia Using Clinical and Treatment Factors: A Retrospective Study With Restricted Cubic Spline and Piecewise Regression Analysis, Frontiers in Public Health. (2026) 14, 10.3389/fpubh.2026.1768432. PMC13144054 42100535

[9] Hasan B. , Bechenati D. , Bethel H. M. et al., A Systematic Review of Length of Stay Linked to Hospital-Acquired Falls, Pressure Ulcers, Central Line-Associated Bloodstream Infections, and Surgical Site Infections, Mayo Clinic Proceedings: Innovations, Quality & Outcomes. (2025) 9, no. 3, 10.1016/j.mayocpiqo.2025.100607. PMC12008517 40256621

[10] Xu S. , Song Z. , Han F. , and Zhang C. , Effect of Appropriate Empirical Antimicrobial Therapy on Mortality of Patients With Gram-Negative Bloodstream Infections: A Retrospective Cohort Study, BMC Infectious Diseases. (2023) 23, no. 1, 10.1186/s12879-023-08329-2. PMC10204198 37221465

[11] Van Heuverswyn J. , Valik J. K. , van der Werff S. D. , Hedberg P. , Giske C. , and Nauclér P. , Association Between Time to Appropriate Antimicrobial Treatment and 30-day Mortality in Patients With Bloodstream Infections: A Retrospective Cohort Study, Clinical Infectious Diseases. (2023) 76, no. 3, 469–478, 10.1093/cid/ciac727. PMC9907509 36065752

[12] Tang F. , Yuan H. , Li X. , and Qiao L. , Effect of Delayed Antibiotic Use on Mortality Outcomes in Patients With Sepsis or Septic Shock: A Systematic Review and Meta-Analysis, International Immunopharmacology. (2024) 129, 10.1016/j.intimp.2024.111616. 38310764

[13] Evans L. , Rhodes A. , Alhazzani W. et al., Surviving Sepsis Campaign: International Guidelines for Management of Sepsis and Septic Shock 2021, Critical Care Medicine. (2021) 49, no. 11, e1063–e1143, 10.1097/CCM.0000000000005337. 34605781

[14] Tamma P. D. , Heil E. L. , Justo J. A. , Mathers A. J. , Satlin M. J. , and Bonomo R. A. , Infectious Diseases Society of America 2024 Guidance on the Treatment of Antimicrobial-Resistant Gram-Negative Infections, Clinical Infectious Diseases. (2024) 10.1093/cid/ciae403. 39108079

[15] Centers for Disease Control and Prevention , Core Elements of Antibiotic Stewardship, 2025, https://www.cdc.gov/antibiotic-use/hcp/core-elements/index.html.

[16] Suissa S. , Immortal Time Bias in Pharmacoepidemiology, American Journal of Epidemiology. (2008) 167, no. 4, 492–499, 10.1093/aje/kwm324. 18056625

[17] Firth D. , Bias Reduction of Maximum Likelihood Estimates, Biometrika. (1993) 80, no. 1, 27–38, 10.1093/biomet/80.1.27.

[18] Koenker R. and Bassett G. , Regression Quantiles, Econometrica. (1978) 46, no. 1, 33–50, 10.2307/1913643.

[19] Cunha A. I. L. , Veronese N. , de Melo Borges S. , and Ricci N. A. , Frailty as a Predictor of Adverse Outcomes in Hospitalized Older Adults: A Systematic Review and Meta-Analysis, Ageing Research Reviews. (2019) 56, 10.1016/j.arr.2019.100960. 31518686

[20] Kim D. H. and Rockwood K. , Frailty in Older Adults, New England Journal of Medicine. (2024) 391, no. 6, 538–548, 10.1056/NEJMra2301292. PMC11634188 39115063

[21] Hoffman T. , Margalit I. , Tabah A. et al., Risk Factors for Mortality Among Older Adults With Hospital-Acquired Bloodstream Infections in the Intensive Care Unit: A Multicenter Cohort Study, Infectious Disease and Therapy. (2025) 14, no. 2, 483–492, 10.1007/s40121-024-01104-z. PMC11829854 39794673

[22] Moon R. C. , MacVane S. H. , David J. , Morton J. B. , Rosenthal N. , and Claeys K. C. , Clinical Outcomes of Early Phenotype-Desirable Antimicrobial Therapy for Enterobacterales Bacteremia, JAMA Network Open. (2024) 7, no. 12, 10.1001/jamanetworkopen.2024.51633. PMC11667351 39714841

[23] Moradi S. , Najafpour Z. , Cheraghian B. , Keliddar I. , and Mombeyni R. , The Extra Length of Stay, Costs, and Mortality Associated With Healthcare-Associated Infections: A Case-Control Study, Health Science Reports. (2024) 7, no. 11, 10.1002/hsr2.70168. PMC11540804 39512247

[24] Kalil A. C. , Metersky M. L. , Klompas M. et al., Management of Adults With Hospital-Acquired and Ventilator-Associated Pneumonia: 2016 Clinical Practice Guidelines by the Infectious Diseases Society of America and the American Thoracic Society, Clinical Infectious Diseases. (2016) 63, no. 5, e61–e111, 10.1093/cid/ciw353. PMC4981759 27418577

[25] Gupta A. B. , Heath M. , Walzl E. et al., Antibiotic De-Escalation in Adults Hospitalized for Community-Onset Sepsis, JAMA Internal Medicine. (2026) 186, no. 2, 192–202, 10.1001/jamainternmed.2025.6919. PMC12723592 41428290

[26] Zhao K. , Zhang Z. , Liang Y. , Wang Y. , and Cai Y. , Effect of Antimicrobial De-Escalation Strategy on 14-Day Mortality Among Intensive Care Unit Patients: A Retrospective Propensity Score-Matched Cohort Study With Inverse Probability-of-Treatment Weighting, BMC Infectious Diseases. (2023) 23, no. 1, 10.1186/s12879-023-08491-7. PMC10401733 37537526

[27] Ohji G. , Doi A. , Yamamoto S. , and Iwata K. , Is De-Escalation of Antimicrobials Effective? A Systematic Review and Meta-Analysis, International Journal of Infectious Diseases. (2016) 49, 71–79, 10.1016/j.ijid.2016.06.002. 27292606

